# Supplementary figures and images for: HLA-A*11:01-restricted CD8+ T cell immunity against influenza A and influenza B viruses in Indigenous and non-Indigenous people
Source: PLoS Pathog. 2022 Mar 7;18(3):e1010337. doi: 10.1371/journal.ppat.1010337 (PMC8929706; doi:10.1371/journal.ppat.1010337)

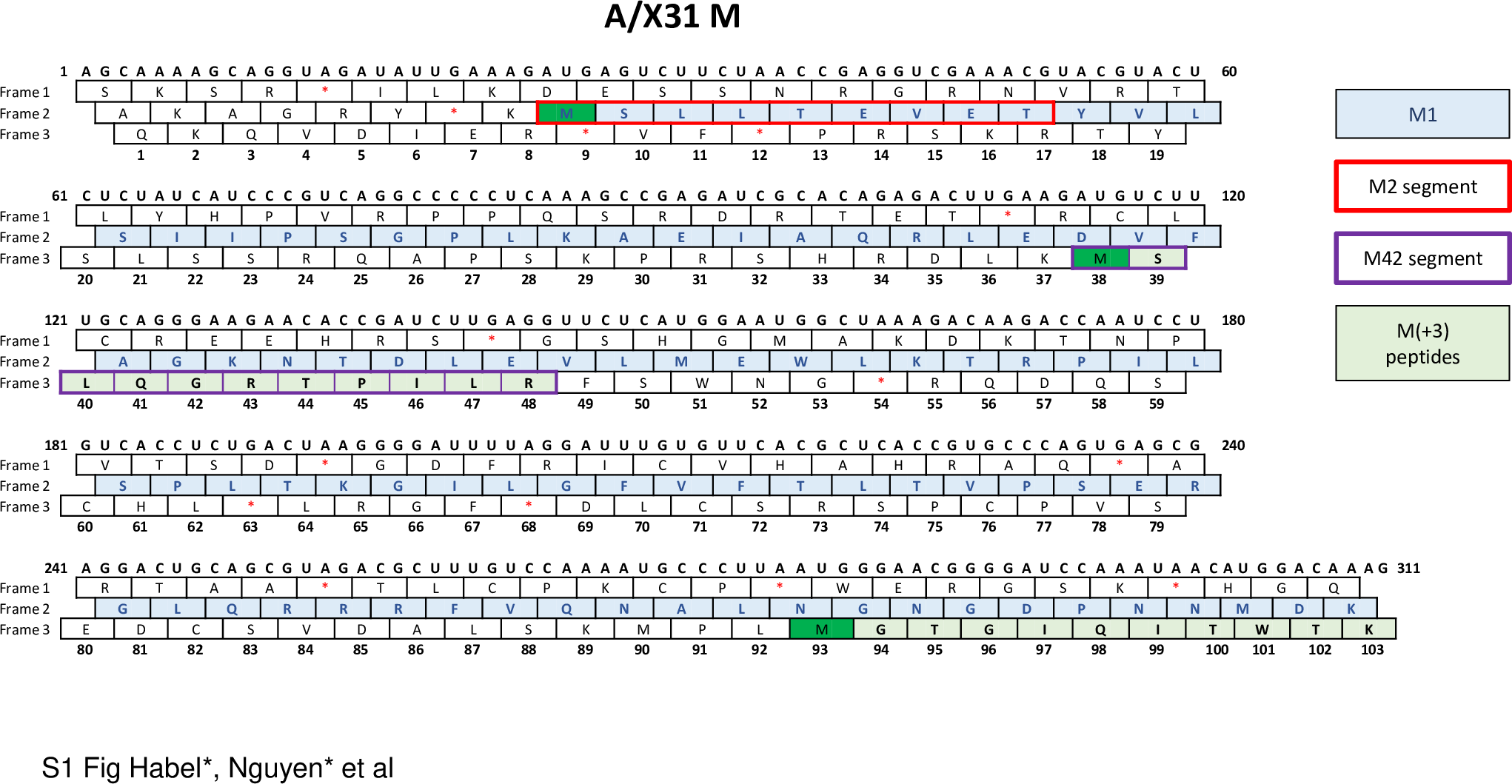

Supplement: S1 Fig — Translation of the 5’ end of A/X31 segment 7 (M) mRNA in three translation frames. The N-terminal portion of M1 is shown in blue with the region also contributing to the N-terminal ectodomain of M2 outlined in red (M2 segment). The alternative ectodomain (M42 segment), encoded in frame 3, is outlined in purple encompassing both an alternative start site and a detected HLA-A*11:01 ligand (M(+3)39-48, light green). A second HLA-A*11:01 ligand encoded in frame 3 (M(+3)94-103, light green), preceded by another potential alternative start site, is also shown. Numbering below relates to translation in frame 3, starting at the viral 5’ UTR. Red asterisks denote stop codons and dark green M are known/proposed start sites. (TIF) [file ppat.1010337.s001.tif]

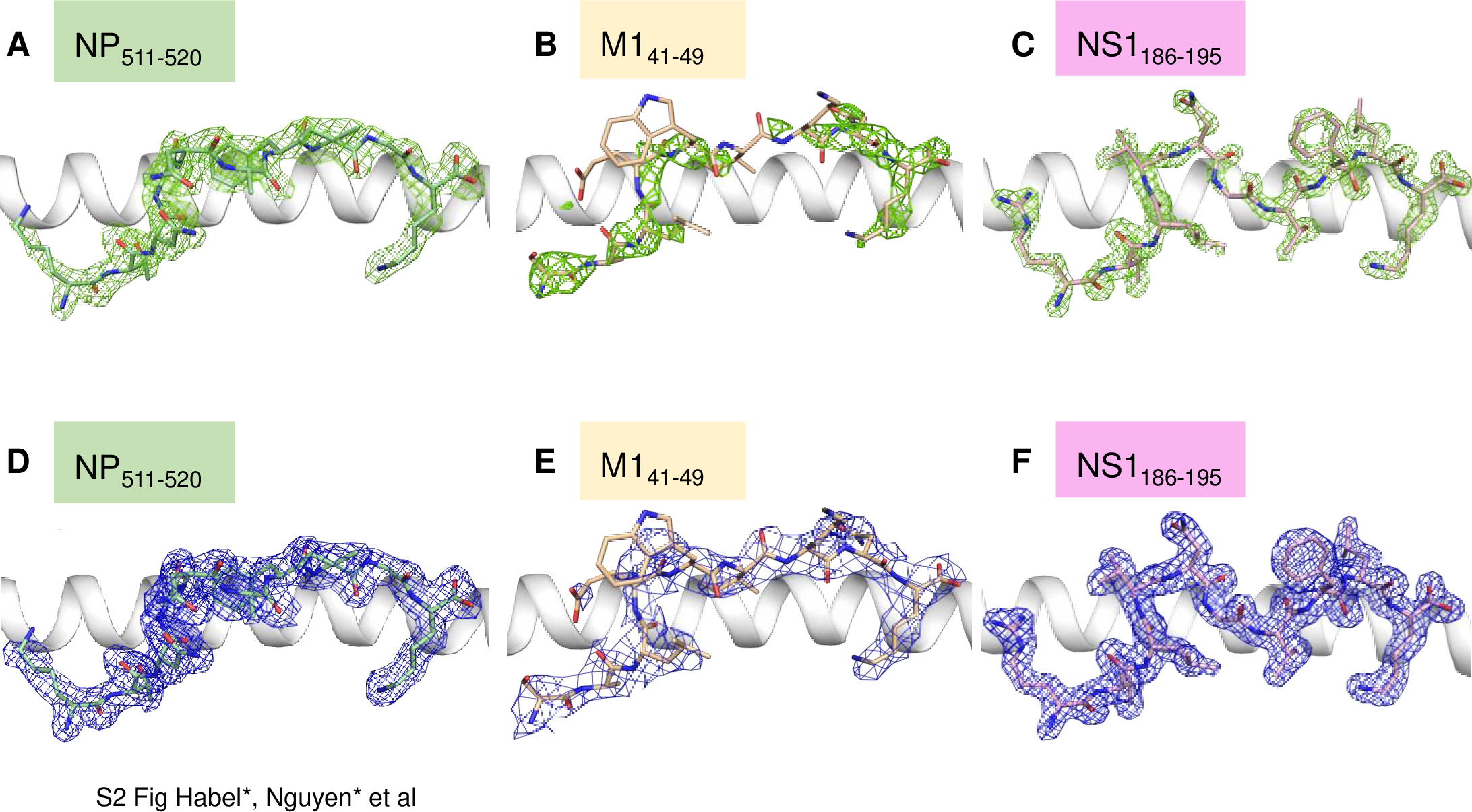

Supplement: S2 Fig — Density map for the structures of the three HLA-A*11:01 (white cartoon) binding to IBV peptides coloured in light green (NP156-166), sand (M141-49) and light pink (NS1186-195). The omit 2Fo-Fc map is contoured at 3 sigma (A-C) or 2.5 sigma (B) and coloured in green on the top panels, while the electron density after refinement is shown by a blue Fo-Fc map contoured at 1 sigma on the bottom panels (D-F). (TIF) [file ppat.1010337.s002.tif]

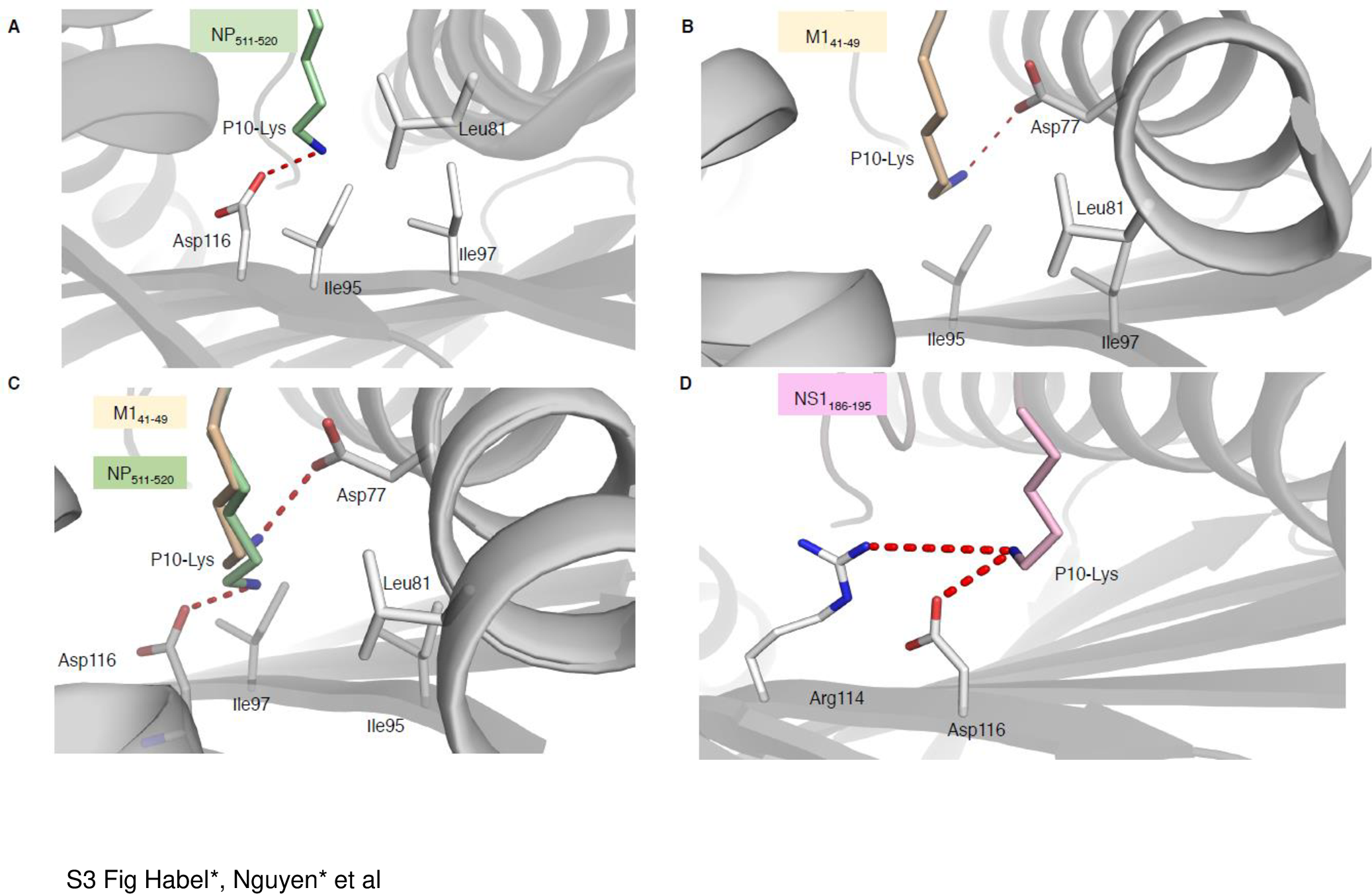

Supplement: S3 Fig — (A) The binding cleft of HLA-A*11:01 (white cartoon) with the peptide NP156-166 (light green stick) interacting with residues within the binding cleft (white stick). (B) The binding cleft of HLA-A*11:01 (white cartoon) with the peptide M141-49 (sand stick) interacting with residues within the binding cleft (white stick). (C) Overlay of HLA-A*11:01 (white cartoon) presenting NP156-166 peptide (light green stick) and HLA-A*11:01 (white cartoon) presenting M141-49 peptide (sand stick) interacting with residues within the binding cleft (white stick). (D) The binding cleft of HLA-A*11:01 (white cartoon) with the peptide NS1186-195 (pink stick) interacting with residues within the binding cleft (white stick). (TIF) [file ppat.1010337.s003.tif]

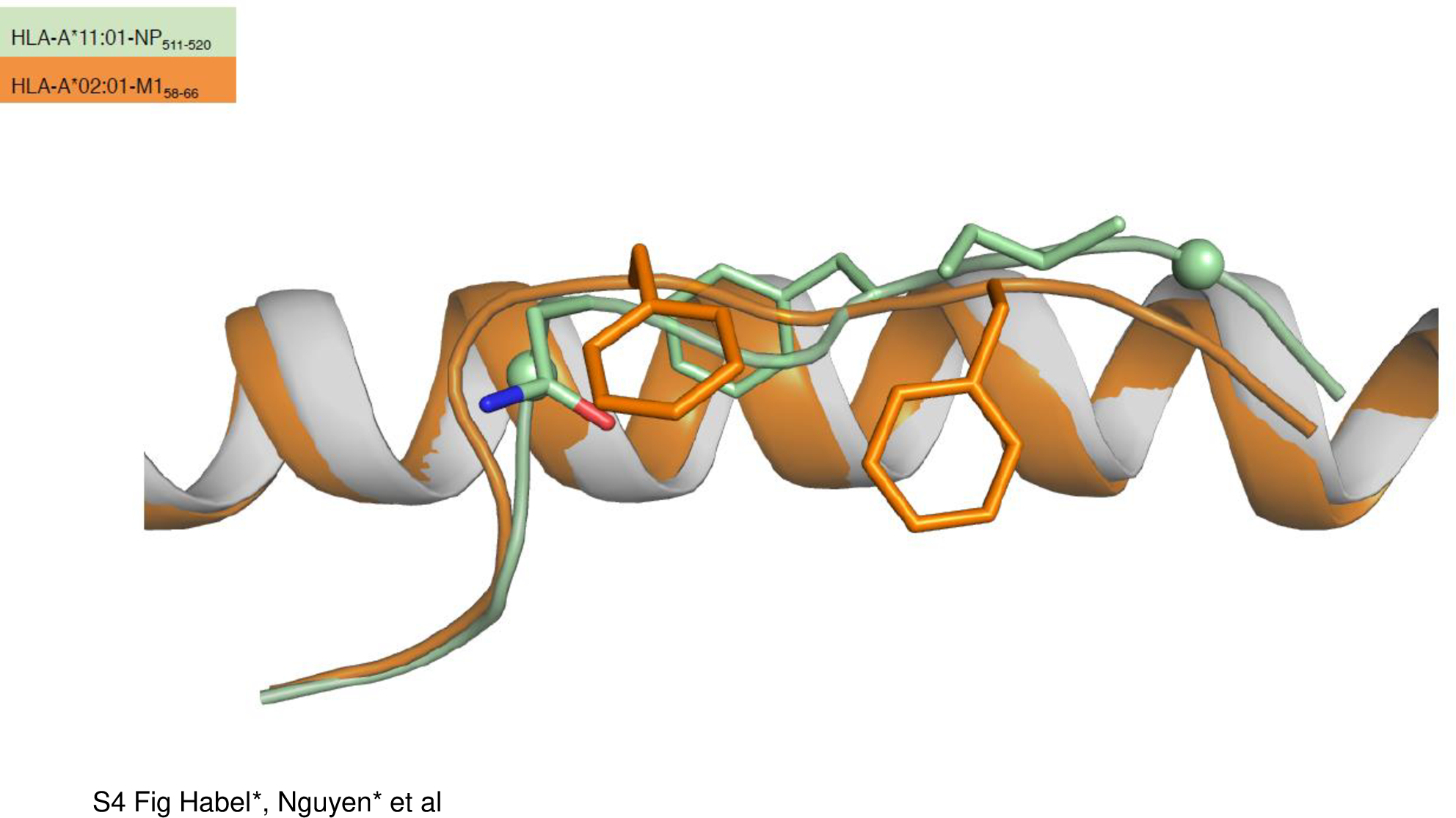

Supplement: S4 Fig — Overlay of HLA-A*11:01 (white cartoon) presenting NP156-166 peptide (light green stick) and HLA-A*02:01 (orange cartoon) presenting M158-66 (orange stick). (TIF) [file ppat.1010337.s004.tif]

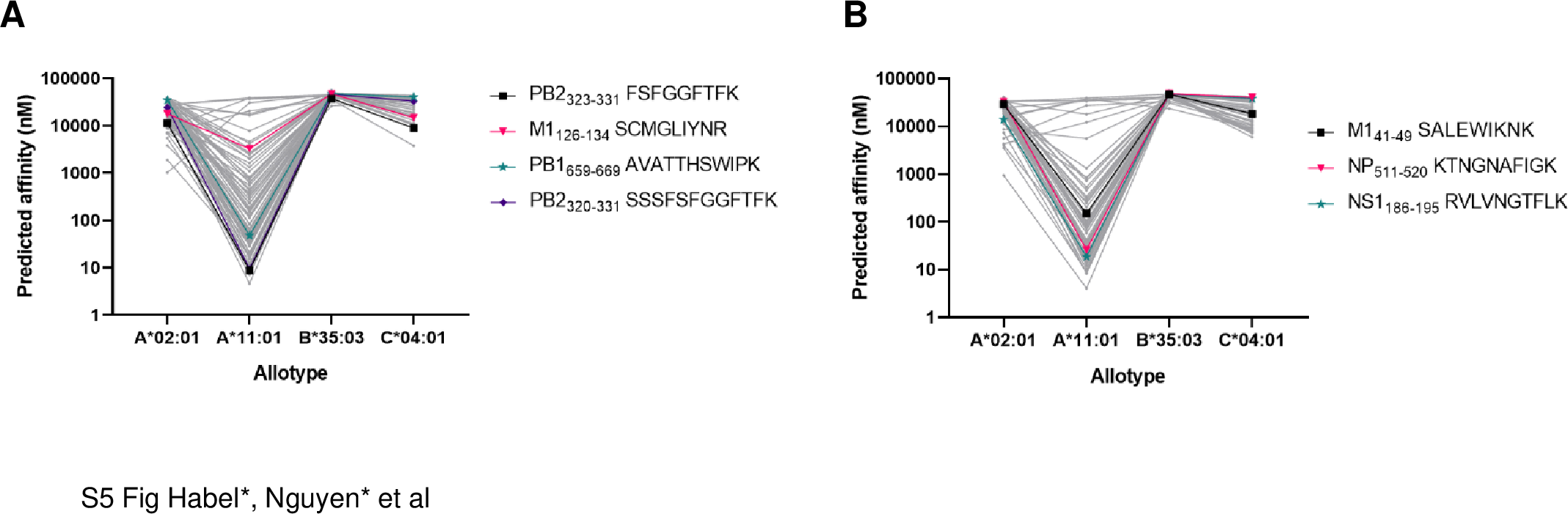

Supplement: S5 Fig — (A-B) Predicted affinity for HLA-A*11:01-restricted (A) IAV-derived and (B) IBV-derived peptides for HLA-A*02:01, A*11:01, B*35:03 and C*04:01, calculated using NetMHC4.0. Each line represents an individual peptide, with immunogenic peptides highlighted by colored lines as shown in the legends. (TIF) [file ppat.1010337.s005.tif]

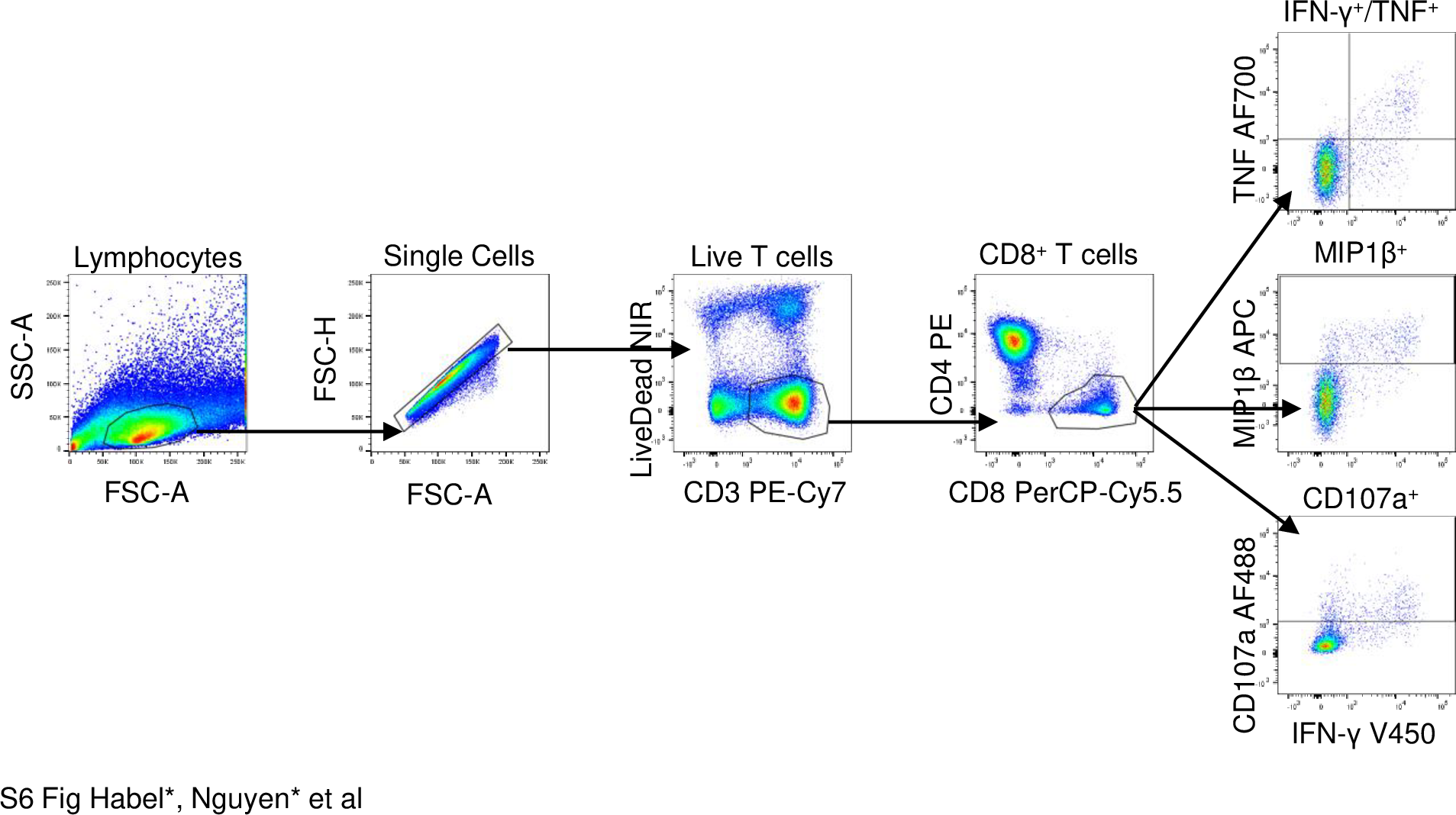

Supplement: S6 Fig — Gating strategy used to determine CD8+ T cell activation and polyfunctionality defined by IFN-γ, TNF, MIP1-β and/or CD107a expression. (TIF) [file ppat.1010337.s006.tif]
