# Supplementary material for: HLA-A*11:01-restricted CD8+ T cell immunity against influenza A and influenza B viruses in Indigenous and non-Indigenous people
Source: PLoS Pathog. 2022 Mar 7;18(3):e1010337. doi: 10.1371/journal.ppat.1010337 (PMC8929706; doi:10.1371/journal.ppat.1010337)
Supplement: S1 Table — (DOCX) [file ppat.1010337.s008.docx]

| **S1 Table. Demographics of Indigenous and non-Indigenous donors** | | | | |
| --- | --- | --- | --- | --- |
| Donor | Age* | Gender | HLA-A | HLA-B |
| **Non-Indigenous** |  |  |  |  |
| NI1 | 47 | n.d. | **11:01**, 31:01 | 44:02, 55:01 |
| NI2 | 20 | n.d. | **11:01**, 03:01 | 38:01, 44:02 |
| NI3 | 48 | n.d. | **11:01**, 01:01 | 14:02, 15:01 |
| NI4 | 60 | n.d. | **11:01**, 02:01 | 35:01, 51:01 |
| NI5 | 47 | n.d. | **11:01**, 02:01 | 44:02, 51:01 |
| NI6 | 46 | F | **11:01**, 01:01 | 08:01, 55:01 |
| NI7 | 22 | M | **11:01**, 31:01 | 40:01, 51:01 |
| NI8 | 48 | M | **11:01**, 23:01 | 15:03, 35:01 |
| NI9 | 34 | M | **11:01**, 26:01 | 07:05, 35:03 |
| NI10 | 73 | F | **11:01**, 02:01 | 40:01 |
| NI11 | 80 | F | **11:01**, 03:01 | 40:01, 44:02 |
| NI12 | 29 | M | **11:01**, 26:01 | 14:01, 27:05 |
| NI13 | 26 | M | **11:01**, 02:01 | 51:01, 55:01 |
| NI14 | 57 | F | **11:01**, 02:01 | 35:01, 44:02 |
| NI15 | 46 | M | **11:01**, 24:02 | 40:01, 56:03 |
| NI16 | 61 | M | **11:01**, 03:01 | 07:02, 18:01 |
| NI17 | 23 | F | **11:01**, 33:03 | 13:01, 58:01 |
| NI18 | n.d. | n.d. | **11:01**, 02:01 | 14:02, 44:02 |
| NI19 | 26 | F | **11:01**, 02:01 | 40:01, 44:02 |
| NI20 | 51 | n.d. | **11:01**, 02:01 | 07:02, 44:02 |
| **Indigenous** |  |  |  |  |
| IN1 | 64 | F | **11:01**, 02:01 | 18:01, 27:04 |
| IN2 | 47 | M | **11:01**, 02:01 | 18:01, 27:04 |
| IN3 | 31 | F | **11:01**, 02:01 | 15:25, 40:02 |
| IN4 | 29 | F | **11:01**, 02:01 | 44:02, 56:01 |
| n.d. no data  *Age in years at time of blood draw | | | | |
