# Supplementary material for: HLA-A*11:01-restricted CD8+ T cell immunity against influenza A and influenza B viruses in Indigenous and non-Indigenous people
Source: PLoS Pathog. 2022 Mar 7;18(3):e1010337. doi: 10.1371/journal.ppat.1010337 (PMC8929706; doi:10.1371/journal.ppat.1010337)
Supplement: S2 Table — (DOCX) [file ppat.1010337.s009.docx]

| **S2 Table.** **Previously reported HLA-A*11:01-restricted peptides from influenza A virus** | | |
| --- | --- | --- |
| **Peptide** | **Sequence** | **Publication** |
| HA_458-467_ | NYKNLYEKVK | Alexander *et al.* (1997) [1] |
| M1_13-21_ | SIIPSGPLK | Alexander *et al.* (1997) [1] |
| M1_125-134_ | ASCMGLIYNR | Alexander *et al.* (2010) [2] |
| M2_45-54_ | RLFFKCIYRR | Alexander *et al.* (2010) [2] |
| NP_407-416_ | SVQPTFSVQR | Alexander *et al.* (2010) [2] |
| NP_413-422_ | SVQRNLPFER | Alexander *et al.* (2010) [2] |
| NS1_122-131_ | AIMDKNVMLK | Alexander *et al.* (2010) [2] |
| NS2_106-115_ | LFEVEQEIRT | Alexander *et al.* (2010) [2] |
| PA_104-113_ | KFLPDLYDYK | Alexander *et al.* (2010) [2] |
| PB1_471-480_ | KLVGINMSKK | Alexander *et al.* (2010) [2] |
| PB1_689-698_ | YQKCCNLFEK | Alexander *et al.* (2010) [2] |
| PB2_322-331_ | SFSFGGFTFK | Alexander *et al.* (2010) [2] |
| PB2_323-332_ | FSFGGFTFKR | Alexander *et al.* (2010) [2] |
| M1_178-187_ | RMVLASTTAK | Assarsson *et al.* (2008) [3] |
| M1_13-21_ | SIIPSGPLK | Chang *et al.* (2013) [4] |
| HA_149-158_ | VTAACSHAGK | Gianfrani *et al.* (2000) [5] |
| HA_450-460_ | RTLDFHDSNVK | Gianfrani *et al.* (2000) [5] |
| HA_63-71_ | GIAPLQLGK | Gianfrani *et al.* (2000) [5] |
| M1_13-21_ | SIIPSGPLK | Gianfrani *et al.* (2000) [5] |
| M1_178-187_ | RMVLASTTAK | Gianfrani *et al.* (2000) [5] |
| M2_70-78_ | KSMREEYRK | Gianfrani *et al.* (2000) [5] |
| NP_342-351_ | RVLSFIKGTK | Gianfrani *et al.* (2000) [5] |
| M1_235-244_ | AYQRMGVQM | Liu *et al.* (2012) [6] |
| NP_188-198_^*^ | TMVMELIRMIK | Liu *et al.* (2016) [7] |
| NP_188-198_^*^ | TIAMELIRMIK | Liu *et al.* (2016) [7] |
| ^*^ Variants of the NP_188-198_ peptide. Amino acid variation is depicted in red font. | | |

1. Alexander J, Oseroff C, Sidney J, Wentworth P, Keogh E, Hermanson G, et al. Derivation of HLA-A11/Kb transgenic mice: functional CTL repertoire and recognition of human A11-restricted CTL epitopes. J Immunol. 1997;159(10):4753-61. Epub 1997/11/20. PubMed PMID: 9366399.

2. Alexander J, Bilsel P, del Guercio MF, Marinkovic-Petrovic A, Southwood S, Stewart S, et al. Identification of broad binding class I HLA supertype epitopes to provide universal coverage of influenza A virus. Hum Immunol. 2010;71(5):468-74. Epub 2010/02/17. doi: 10.1016/j.humimm.2010.02.014. PubMed PMID: 20156506; PubMed Central PMCID: PMCPMC2856764.

3. Assarsson E, Bui HH, Sidney J, Zhang Q, Glenn J, Oseroff C, et al. Immunomic analysis of the repertoire of T-cell specificities for influenza A virus in humans. J Virol. 2008;82(24):12241-51. Epub 2008/10/10. doi: 10.1128/jvi.01563-08. PubMed PMID: 18842709; PubMed Central PMCID: PMCPMC2593359.

4. Chang CX, Tan AT, Or MY, Toh KY, Lim PY, Chia AS, et al. Conditional ligands for Asian HLA variants facilitate the definition of CD8+ T-cell responses in acute and chronic viral diseases. Eur J Immunol. 2013;43(4):1109-20. Epub 2013/01/03. doi: 10.1002/eji.201243088. PubMed PMID: 23280567; PubMed Central PMCID: PMCPMC3655610.

5. Gianfrani C, Oseroff C, Sidney J, Chesnut RW, Sette A. Human memory CTL response specific for influenza A virus is broad and multispecific. Hum Immunol. 2000;61(5):438-52. Epub 2000/04/25. doi: 10.1016/s0198-8859(00)00105-1. PubMed PMID: 10773346.

6. Liu J, Zhang S, Tan S, Yi Y, Wu B, Cao B, et al. Cross-allele cytotoxic T lymphocyte responses against 2009 pandemic H1N1 influenza A virus among HLA-A24 and HLA-A3 supertype-positive individuals. J Virol. 2012;86(24):13281-94. Epub 2012/09/26. doi: 10.1128/JVI.01841-12. PubMed PMID: 23015716.

7. Liu WJ, Tan S, Zhao M, Quan C, Bi Y, Wu Y, et al. Cross-immunity Against Avian Influenza A(H7N9) Virus in the Healthy Population Is Affected by Antigenicity-Dependent Substitutions. J Infect Dis. 2016;214(12):1937-46. Epub 2016/10/16. doi: 10.1093/infdis/jiw471. PubMed PMID: 27738054.
