# Supplementary material for: HLA-A*11:01-restricted CD8+ T cell immunity against influenza A and influenza B viruses in Indigenous and non-Indigenous people
Source: PLoS Pathog. 2022 Mar 7;18(3):e1010337. doi: 10.1371/journal.ppat.1010337 (PMC8929706; doi:10.1371/journal.ppat.1010337)
Supplement: S3 Table — (DOCX) [file ppat.1010337.s010.docx]

| **S3 Table. HLA-A*11:01-restricted IAV peptides identified by mass spectrometry.** | | | |
| --- | --- | --- | --- |
| **Peptide** | **Sequence** | **Affinity (nM)*** | **Peptide pool** |
| PB1-F2 44-53 | KTMNQVVMPK | 4.3 | A |
| PB2 323-331 | FSFGGFTFK | 6.3 | A |
| PB1(+2) 607-616 | TTLEISTFLK | 7.3 | A |
| PB2 320-331 | SSSFSFGGFTFK | 8.7 | A |
| PB1-F2 45-53 | TMNQVVMPK | 10.6 | A |
| PB2 473-482 | MSMRGVRISK | 11.4 | A |
| PA(+2) 57-65 | QIFTSSMSK | 11.5 | A |
| M1 179-187 | MVLASTTAK | 14.4 | A |
| PB2 128-140 | GTFGPVHFRNQVK | 14.5 | A |
| NP 189-198 | MVMELVRMIK | 15.1 | A |
| NA 30-38 | VTTVTLHFK | 15.1 | B |
| PB1 523-531 | MSIGVTVIK | 15.4 | B |
| PB2 565-574 | SQNPTMLYNK | 15.8 | B |
| NP 22-31 | ATEIRASVGK | 18 | B |
| PA(+2) 56-65 | IQIFTSSMSK | 21.2 | B |
| PB1-F2 12-20 | STGHISTQK | 22.2 | B |
| M(+3) 94-103 | GTGIQITWTK | 23.6 | B |
| PB1 659-669 | AVATTHSWIPK | 26.9 | B |
| PB1 337-347 | LSIAPIMFSNK | 30.8 | B |
| PB2 105-116 | ITNTVHYPKIYK | 32.8 | B |
| NP 342-351 | RVLSFIKGTK | 32.9 | C |
| NP 462-470 | GVFELSDEK | 33.5 | C |
| PB1 643-653 | AVMMPAHGPAK | 34.9 | C |
| PB1(+3) 705-714 | KILPQQFIQK | 40.4 | C |
| NS1 205-217 | SSNENGRPPLTPK | 40.6 | C |
| NS2 7-18 | SSFQDILLRMSK | 41.1 | C |
| M1 12-21 | LSIIPSGPLK | 45 | C |
| PB2 116-126 | KTYFERVERLK | 51.8 | C |
| PB2 689-699 | AVLRGFLILGK | 54.8 | C |
| NP 165-174 | SLMQGSTLPR | 59.1 | D |
| NP 187-195 | GTMVMELVR | 83.3 | D |
| PB2 23-33 | TTVDHMAIIKK | 87.4 | D |
| NS2 30-39 | GMITQFESLK | 89.4 | D |
| PB1 748-757 | STIEELRRQK | 94.6 | D |
| PB1 422-433 | SILNLGQKRYTK | 94.8 | D |
| PB2 741-752 | SSILTDSQTATK | 101.1 | D |
| NS1 62-70 | KQIVERILK | 110.2 | D |
| M1 47-57 | KTRPILSPLTK | 111.5 | D |
| PB1(+3) 9-18 | GCQSDLTFLK | 113.6 | D |
| PB2 175-187 | RILTSESQLTITK | 120.5 | E |
| PB1 180-188 | GITTHFQRK | 125.7 | E |
| NS1 121-131 | QAIMDKNIILK | 186.7 | E |
| NS2 5-15 | TVSSFQDILLR | 208.4 | E |
| PB2 494-503 | VVVSIDRFLR | 235 | E |
| PA 61-73 | IIVELGDPNALLK | 274 | E |
| NP 413-422 | SVQRNLPFDR | 281.4 | E |
| M1 243-252 | RMGVQMQRFK | 302 | E |
| M1 244-252 | MGVQMQRFK | 957.6 | E |
| PB2 709-721 | SINELSNLAKGEK | 309.9 | E |
| PB1(+3) 12-18 | SDLTFLK | 319.8 | E |
| PB1 366-379 | TQIPAEMLASIDLK | 364.5 | F |
| NP 314-325 | SLIRPNENPAHK | 398.1 | F |
| PB1 113-121 | VVQQTRVDK | 419.7 | F |
| NP 263-273 | ALILRGSVAHK | 428.7 | F |
| PA(+3) 30-40 | RVWGGPENRNK | 469 | F |
| PB1 727-737 | RIDFESGRIKK | 507.4 | F |
| PB2 682-692 | GTAGVESAVLR | 666.1 | F |
| NP 27-38 | ASVGKMIGGIGR | 698.1 | F |
| PB1-F2 49-59 | VVMPKQIVYWR | 742.7 | F |
| PA 12-22 | MIVELAEKTMK | 761.8 | F |
| M(+3) 39-48 | SLQGRTPILR | 869.4 | G |
| PB2 608-618 | GTFDTAQIIKL | 1503.8 | G |
| PA(+2) 58-65 | IFTSSMSK | 1832.4 | G |
| PB1 1-11 | MDVNPTLLFLK | 2077.3 | G |
| M2 58-70 | GLKGGPSTEGVPK | 2827.2 | G |
| M1 168-178 | TTNPLIRHENR | 4986.2 | G |
| PB1 269-279 | SGLPVGGNEKK | 5412.8 | G |
| PB1(+2) 694-705 | NKCTKGAAIYLK | 6002.3 | G |
| PB1(+2) 697-705 | TKGAAIYLK | 6075.6 | G |
| M1 92-100 | NMDKAVKLY | 12384.6 | G |
| PB1 491-498 | EFTSFFYR | 13009.7 | H |
| M1 229-242 | LKNDLLENLQAYQK | 16576.6 | H |
| PB2 273-281 | SADPLASLL | 26068.7 | H |
| PA 476-483 | AMDDFQLI | 31417.9 | H |
| NP 70-79 | AFDERRNKYL | 35891.7 | H |
| PA 204-211 | RFEITGTM | 37637.2 | H |
| M1 215-230 | AMRTIGTHPSSSAGLK | 38957.7 | H |
| NS1 150-157 | FTEEGAIV | 41997.4 | H |
| PB1(+3) 55-65 | SVLRKGKMDNK | 319.8 | H |
| *Binding affinities predicted by NetMHCpan 4.0 | | | |
