# Supplementary material for: HLA-A*11:01-restricted CD8+ T cell immunity against influenza A and influenza B viruses in Indigenous and non-Indigenous people
Source: PLoS Pathog. 2022 Mar 7;18(3):e1010337. doi: 10.1371/journal.ppat.1010337 (PMC8929706; doi:10.1371/journal.ppat.1010337)
Supplement: S4 Table — (DOCX) [file ppat.1010337.s011.docx]

| **S4 Table. HLA-A*11:01-restricted IBV peptides identified by mass spectrometry.** | | | |
| --- | --- | --- | --- |
| **Peptide** | **Sequence** | **Affinity (nM)*** | **Peptide pool** |
| PB2 164-172 | VIMEILFPK | 5.1 | 1 |
| PB2 115-124 | RVYESFFLRK | 6.5 | 1 |
| NP 226-235 | STFAGSTLPR | 7.1 | 1 |
| PB2 115-123 | RVYESFFLR | 9.2 | 1 |
| NP 332-341 | VVLPISIYAK | 9.3 | 1 |
| NP 36-44 | ATLAPPSNK | 10.4 | 1 |
| BM2 54-62 | SILRHSYQK | 10.8 | 1 |
| NA 247-255 | GVSECRFLK | 11.1 | 1 |
| NS1 186-195 | RVLVNGTFLK | 11.1 | 1 |
| NS2 22-31 | STHSSSVLMK | 12.1 | 1 |
| M1 236-246 | SSMGNSALVKK | 16.1 | 1 |
| NP 511-520 | KTNGNAFIGK | 16.3 | 1 |
| HA 261-269 | IVVDYMVQK | 18.2 | 1 |
| PB2 594-602 | SGFARAVLK | 18.4 | 1 |
| PA 365-374 | ATGDGLTYQK | 19.1 | 1 |
| NA 31-39 | LLYSDILLK | 20.9 | 1 |
| NP 171-183 | VTFLKEEVKTMYK | 20.9 | 1 |
| NS1 191-201 | GTFLKHPNGYK | 24.9 | 1 |
| M1 187-201 | KTMNGMGKGEDVQKL | 28.6 | 1 |
| NP 540-549 | QTIPNFFFGR | 31.3 | 2 |
| PB1 113-121 | ALMVTTVDK | 32.5 | 2 |
| PB2 146-154 | RVLLNPLTK | 35.5 | 2 |
| NA 215-223 | HSYANNILR | 38.3 | 2 |
| NS2 8-16 | TQIEWRMKK | 38.8 | 2 |
| NP 77-85 | KSVYNMVVK | 43.5 | 2 |
| NP 322-331 | VVVRPSVASK | 51.9 | 2 |
| PB2 135-145 | ITFGPVERVRK | 55.5 | 2 |
| HA 393-401 | GVAVAADLK | 56.5 | 2 |
| PA 543-551 | SLFVSGREK | 75 | 2 |
| M1 41-49 | SALEWIKNK | 92.3 | 2 |
| PA 618-626 | GTQEGKLVK | 98.2 | 2 |
| HA 402-412 | STQEAINKITK | 101.2 | 2 |
| HA 43-53 | GVIPLTTTPTK | 109.4 | 2 |
| HA 274-287 | GTITYQRGILLPQK | 119.8 | 2 |
| PB2 25-35 | TTVDQYNIIRK | 163.3 | 2 |
| PB2 608-617 | EVMKTDQFIK | 220.6 | 2 |
| M1 235-245 | QSSMGNSALVK | 236.5 | 2 |
| M1 210-220 | GVLRSLGASQK | 245 | 2 |
| NA 192-203 | GVDGPDNNALLK | 398.5 | 3 |
| NS2 33-41 | IQSQFEQLK | 460.1 | 3 |
| NP 55-68 | TTISEADVGRKTQK | 461.9 | 3 |
| NP 121-131 | ATDDKKTEFQK | 586 | 3 |
| NP 203-211 | QMNDVCFQR | 850.7 | 3 |
| NS2 76-84 | RIDDNILFH | 1006.2 | 3 |
| HA 410-423 | ITKNLNSLSELEVK | 1235.5 | 3 |
| NP 167-175 | SPIRVTFLK | 1237.5 | 3 |
| NA 4-12 | STIQTLTLF | 2569.5 | 3 |
| PB1 142-150 | ALNTTITSF | 12238.6 | 3 |
| NS1 16-23 | ATNATINF | 18062.3 | 3 |
| NP 392-400 | AAYEDLRVL | 27131.8 | 3 |
| M1 217-234 | ASQKNGEGIAKDVMEVLK | 29975.7 | 3 |
| HA 518-529 | TFDAGEFSLPTF | 33146.8 | 3 |
| PB2 277-285 | NPLELAVEI | 35520.4 | 3 |
| NP 79-86 | VYNMVVKL | 37906.9 | 3 |
| NP 12-22 | GTIDKAPEEIT | 37980 | 3 |
| HA 220-228 | YGDSKPQKF | 39066.1 | 3 |
| BM2 83-97 | DHIIIEGLSAEEIIK | 41636.8 | 3 |
| *Binding affinities predicted by NetMHCpan 4.0 | | | |
