# Supplementary material for: HLA-A*11:01-restricted CD8+ T cell immunity against influenza A and influenza B viruses in Indigenous and non-Indigenous people
Source: PLoS Pathog. 2022 Mar 7;18(3):e1010337. doi: 10.1371/journal.ppat.1010337 (PMC8929706; doi:10.1371/journal.ppat.1010337)
Supplement: S5 Table — (DOCX) [file ppat.1010337.s012.docx]

| **S5 Table. Thermal stability of peptide-HLA-A*11:01 complexes.** | | |
| --- | --- | --- |
| **Virus** | **pHLA-A*11:01 complex** | **Tm (^◦^C)** |
| IBV | M1_41-49_ (SALEWIKNK) | 57.60 ± 0.10 |
|  | NP_511-520_ (KTNGNAFIGK) | 61.30 ± 0.25 |
|  | NS1_186-195_ (RVLVNGTFLK) | 61.30 ± 0.50 |
| IAV | PB2_320-331_ (SSSFSFGGFTFK) | 50.50 ± 0.10 |
|  | PB2_323-331_ (FSFGGFTFK) | 53.80 ± 0.05 |
|  | PB1_659-669_ (AVATTHSWIPK) | 59.20 ± 0.25 |
| Tm is the thermal midpoint temperature, experiment done in duplicate at two concentrations (n=1), error are S.E.M. | | |
