# Supplementary material for: HLA-A*11:01-restricted CD8+ T cell immunity against influenza A and influenza B viruses in Indigenous and non-Indigenous people
Source: PLoS Pathog. 2022 Mar 7;18(3):e1010337. doi: 10.1371/journal.ppat.1010337 (PMC8929706; doi:10.1371/journal.ppat.1010337)
Supplement: S6 Table — (DOCX) [file ppat.1010337.s013.docx]

| **S6 Table. Data collection and refinement statistics.** | | | |
| --- | --- | --- | --- |
| **Data Collection Statistics** | **HLA-A*11:01- NP_511-520_** | **HLA-A*11:01- M1_41-49_** | **HLA-A*11:01-NS1_186-195_** |
| Space group | **P 4_3_** | **P 2_1_ 2_1_ 2** | **P 2_1_** |
| Cell Dimensions (a,b,c) (Å) | 119.48, 119.48, 63.46 | 71.33, 120.41, 122.33 | 50.875, 82.262, 57.027 |
| Resolution (Å) | 43.50 – 2.08  (2.14 – 2.08) | 46.43 – 2.95  (3.13 – 2.95) | 46.82 – 1.82  (1.86 – 1.82) |
| Total number of observations | 745179 (48206) | 76936 (12776) | 265223 (15947) |
| Number of unique observations | 53875 (3912) | 21329 (3472) | 38748 (2267) |
| Multiplicity | 13.8 (12.3) | 3.6 (3.7) | 6.8 (7.0) |
| Data completeness (%) | 99.5 (93.5) | 93.8 (96.1) | 99.9 (100) |
| I/σ_I_  Mn(I) half-set correlation CC(1/2) | 11.1 (2.0)  0.997(0.589) | 10.1 (2.0)  0.993(0.791) | 9.9 (1.9)  0.997 (0.682) |
| R_p.i.m_^a^ (%) | 7.1 (59.6) | 7.8 (38.0) | 7.3 (69.9) |
| **Refinement Statistics** |  |  |  |
| Non-hydrogen atoms | 6839 | 6360 | 3404 |
| Protein | 6312 | 6329 | 3174 |
| Water | 526 | 31 | 230 |
| R*factor*^b^ (%) | 0.173 | 0.226 | 0.194 |
| *R_free_*^b^ (%) | 0.217 | 0.278 | 0.225 |
| Rms deviations from ideality |  |  |  |
| Bond lengths (Å) | 0.010 | 0.007 | 0.010 |
| Bond angles (°) | 1.05 | 0.95 | 1.04 |
| Ramachandran plot (%) |  |  |  |
| Allowed region | 99.0 | 95.0 | 99.0 |
| Generously allowed region | 1.0 | 4.0 | 1.0 |
| Disallowed region | 0 | 0 | 0 |
| ^a^R_p.i.m_ = Σ_hkl_ [1/(N-1)]^1/2^ Σ_i_ \| I_hkl, i_ - <I_hkl_> \| / Σ_hkl_ <I_hkl_>  ^b^ R_factor_ = Σ_hkl_ \| \| F_o_ \| - \| F_c_ \| \| / Σ_hkl_ \| F_o_ \| for all data except ≈ 5% which were used for R_free_ calculation. Values in parentheses are for the highest resolution shell. | | | |
