# Supplementary material for: HLA-A*11:01-restricted CD8+ T cell immunity against influenza A and influenza B viruses in Indigenous and non-Indigenous people
Source: PLoS Pathog. 2022 Mar 7;18(3):e1010337. doi: 10.1371/journal.ppat.1010337 (PMC8929706; doi:10.1371/journal.ppat.1010337)
Supplement: Attachment — Submitted filename: Habel et al A11 CD8 T cells rebuttal letter_resubmission.docx [file ppat.1010337.s015.docx]

RESPONSE TO REVIEWERS’ COMMENTS

We thank the Reviewers for their highly favourable comments, which have allowed us to improve the manuscript and further clarify our findings. We also thank the Editors for giving us the opportunity of submitting the revised version of our manuscript.

We have performed all the additional analyses recommended by the Reviewers (depicted in modified Figures 2, 4, 5, 7 and Supplementary Figure 5). Additional CD8^+^ T cell expansions and intracellular cytokine staining experiments were conducted for influenza A virus peptide stimulations (Figures 2 and 4) to include an additional four HLA-A*11:01-expressing individuals (added to Supplementary Table 1).

We have also modified our analysis approach to more clearly define our requirements for determining immunogenicity. We have also discussed all the major points brought to our attention by the Reviewers.

Specific responses to the Reviewers’ comments are outlined in a point-by point form (and presented in blue).

Reviewer's Responses to Questions

Part I – Summary

Reviewer #1: In this manuscript, Habel et al. aim at characterizing HLA-A*11:01-restricted CD8+ T cell responses in HLA-A*11:01-expressing Indigenous and non-Indigenous Australian donors. To this end, the authors used an experimental approach, which combined mass spectrometry analysis to identify peptides expressed by HLA-A*11:01 and in vitro T cell stimulation for testing their immunogenicity. Although the results of this analysis may help increase our knowledge of the CD8+ T cell response against Influenza viruses, data provided in the study are limited (especially for IAV reactivity) and do not allow to make general conclusion on immunodominant epitopes involved in potentially protective CD8+ immune response in HLA-A*11:01+ individuals. Overall, developing a universal influenza vaccine based on this evidence would be quite ambitious.

We thank the Reviewer for their comments. Following the Reviewer’s comment, we have extended our cohort of HLA-A*11:01 individuals to n=24 for our study. We have also addressed the immunodominance question as described below.

Reviewer #2: This is an interesting and well-performed study of HLA-A*11:01-restricted CD8+ T cell responses to influenza A and B virus peptides. HLA-A*11:01 is an allele prevalent in East Asia and Oceania, and highly expressed in indigenous populations at high risk of influenza infection. Current influenza vaccines are designed primarily to induce neutralizing antibodies against hemagglutinin and neuraminidase. Improving the efficacy of influenza A and B vaccines may be achieved by targeting and activating influenza-specific cytotoxic CD8+ T cells and memory formation.

The authors used Masspec to identify influenza A- or B-derived peptides eluted from HLA class I after infection of APCs in vitro. 79 influenza A and 57 influenza B derived peptides were subsequently screened for immunogenicity in HLA-A*11:01-positive PBMC assays. The authors identified three immunodominant influenza A- and three influenza B-derived peptides from PB1, PB2, M, NS1 and NP proteins that appeared highly conserved among influenza viruses.

The study also includes data on structures of HLA-A*11:01 in complex with influenza B-derived peptides identified. Since this lies outside of my field of expertise, I will not comment on this part of the study.

This study's results may inform the design of future influenza vaccines more suitable to meet the needs of indigenous populations, but also the design of more effective influenza vaccines in general, by focusing on viral proteins distinct from hemagglutinin or neuraminidase, targeted by CD8+ T cells.

We thank the Reviewer for their comments.

Part II – Major Issues: Key Experiments Required for Acceptance

Please use this section to detail the key new experiments or modifications of existing experiments that should be absolutely required to validate study conclusions.

Generally, there should be no more than 3 such required experiments or major modifications for a "Major Revision" recommendation. If more than 3 experiments are necessary to validate the study conclusions, then you are encouraged to recommend "Reject".

Reviewer #1: Given the variability in the T cell responses and the low number of donors analyzed, this reviewer believes that including more donors in the screenings shown in Fig. 2 and 4 may help for a better definition of statistical significance in immunodominant epitopes presented on HLA-A*11:01 and targeted by CD8+ T cells in HLA-A*11:01+ donors.

Conclusions regarding immunodominance are made on very few donors (e.g. 2 out of 4 donors, Fig. 2A; 1 out of 3 donors, Fig. 2B etc.), thus resulting limited and potentially biased due to the low number of donors tested.

We thank the Reviewer for their suggestion. To better understand the HLA-A*11:01-restricted CD8^+^ T cell response to influenza A virus-derived peptides, we performed additional CD8^+^ T cell expansions and intracellular cytokine staining experiments in four additional HLA-A*11:01^+^ donors, for a total of n=7 for these figures. Figures 2A, 2B, 2D and 4A-D have been updated to include the additional four donors in the analyses. Overall, additional of the HLA-A*11:01-expressing participants confirmed our previous findings. Additionally, as more HLA-A*11:01-positive donors are included in our datasets, we altered our polyfunctionality analysis to incorporate a permutations test to determine significant differences in polyfunctionality after differential peptide or DMSO (negative control) stimulation.

The additional four HLA-A*11:01 donors bring our cohort of HLA-A*11:01-expressing individuals to 24. We hope the Reviewer appreciates that based on the global distribution of HLA-A*11:01 being at ~7.3%, we needed to HLA type ~328 participants at $AUD 220 (total of $AUD 72,160) for our study.

We have modified the Figures, Results, Figure Legends and Methods accordingly to reflect additional HLA-A*11:01-expressing participants.

Reviewer #2: Main points:

C1R cells express endogenous HLA-C*04:01, so eluted peptides may bind to this allele rather than the transduced HLA-A*11:01. The data is corrected by subtracting peptides identified in previous studies using similar technology. This limitation is clearly mentioned in the manuscript already.

Another limitation concerns the low number of PBMC samples stimulated with a given peptide in screening experiments testing PBMC peptide reactivity, derived from 3 individuals per peptide only. This may have reduced the number of peptides identified, and could also have introduced false positive results, particularly since all 4 indigenous donors were also positive for HLA-A*02:01. Identified peptides were tested with more PBMC samples in the experiment shown in figure 7, but also there the possibility that responses to the identified “HLA-A*11:01” peptides were actually caused by presentation on HLA-A*02:01 is not excluded. This issue could be addressed by stimulating donor PBMCs with the identified peptides presented by C1R cells transduced either with HLA-A*11:01 or -A*02:01.

We thank the Reviewer for their thoughtful comments. As stated above in our response to Reviewer 1, we have increased our sample size for IAV peptide screening to now have n=7 donors. Including these additional HLA-A*11:01-expressing donors has confirmed our previous findings, which depicted a low number of responders to previously reported HLA-A*11:01- or -A*03 supertype-restricted IAV peptides (Figure 2A) as well as our immunopeptidomic-identified peptides (Figure 4B,C).

While the PBMCs in the experiment shown in Figure 7 were initially stimulated with virus-infected autologous PBMCs, they were restimulated by peptide-pulsed C1R cells transduced with either HLA-A*11:01 or -A*02:01 to isolate the presenting HLA allotype. This gives us confidence that the observed CD8^+^ T cell responses observed to the 7 immunogenic HLA-A*11:01-restricted epitopes are indeed restricted to HLA-A*11:01 in this assay.

HLA-A*11:01 restriction across all prior assays is supported by the highly dissimilar peptide binding preferences of HLA-A*02:01 and HLA-A*11:01. Whilst HLA-A*11:01 favours peptides possessing Val, Ser and Thr at P2 and basic (Arg and Lys) residues at the C-terminus (Figure 3B), HLA-A*02:01 favours those possessing Leu and Met at P2 and hydrophobic (Val and Leu) residues at the C-terminus (1). As such the peptides anticipated to bind these two allotypes are highly distinct, with the majority of the peptides tested here (including the 7 immunogenic peptides) predicted to bind HLA-A*11:01 than HLA-A*02:01, as calculated by NetMHC4.0 (2, 3) (Response Figure 1).

*Response Figure 1: Predicted affinity of tested peptides from IAV (left) and IBV (right) for HLA-A*02:01, -A*11:01, -B*35:03 and -C*04:01. Each line represents an individual peptide, with immunogenic peptides highlighted by coloured lines as shown in the legends. Predicted affinities were calculated using NetMHC4.0.*

Moreover, none of the immunogenic IBV derived peptides (M1_41-49_ SALEWIKNK, NP_511-520_ KTNGNAFIGK or NS1_186-195_ RVLVNGTFLK) were identified in our previous analysis of HLA-A*02:01 presentation during IBV infection using the same B/Malaysia strain (1). Nor were the immunodominant IAV peptides isolated on analysis of HLA-A*02:01 presentation during IAV infection (unpublished data) or reported in the Immune epitope database as HLA-A*02:01 ligands ([www.iedb.org](http://www.iedb.org/), accessed 06.12.2021).

While it is possible that enzymatic processing of these peptides might generate improved binders for HLA-A*02:01 (as compared to the full-length sequences), assessment with NetMHC4.0 did not identify any shorter sequences within the immunodominant peptides that were predicted to bind HLA-A*02:01. We therefore have no evidence that the 7 HLA-A*11:01-restricted immunodominant epitopes are presented by HLA-A*02:01 during infection, suggesting it is unlikely that the responses observed against these peptides are restricted to HLA-A*02:01.

Following the Reviewer’s comment, we included the Response Figure 1 as Supplementary Figure 5 and added the following statements to Results (page 12):

“While the PBMCs were initially stimulated with virus-infected autologous PBMCs, they were restimulated by peptide-pulsed C1R cells transduced with either HLA-A*11:01 or -A*02:01 to isolate the presenting HLA allotype, confirming the observed CD8^+^ T cell responses observed to the seven immunogenic HLA-A*11:01-restricted epitopes are indeed restricted to HLA-A*11:01 in our assay.

HLA-A*11:01 restriction across all prior assays is supported by the highly dissimilar peptide binding preferences of HLA-A*02:01 and HLA-A*11:01. Whilst HLA-A*11:01 favours peptides possessing Val, Ser and Thr at P2 and basic (Arg and Lys) residues at the C-terminus (Figure 3B), HLA-A*02:01 favours those possessing Leu and Met at P2 and hydrophobic (Val and Leu) residues at the C-terminus (29). As such the peptides anticipated to bind these two allotypes are highly distinct, with the majority of the peptides tested here (including the 7 immunogenic peptides) exhibiting lower predicted nM affinity for HLA-A*11:01, as compared to HLA-A*02:01 as calculated by NetMHC4.0 (30, 31) (Supplementary Figure 5). Moreover, while it is possible that enzymatic processing of these peptides might generate improved binders for HLA-A*02:01 (as compared to the full-length sequences), assessment with NetMHC4.0 did not identify any shorter sequences within the immunogenic peptides that were predicted to bind HLA-A*02:01. We therefore have no evidence that the 7 HLA-A*11:01-restricted immunogenic peptides are presented by HLA-A*02:01 during infection, suggesting it is unlikely that the responses observed against these peptides are restricted to HLA-A*02:01.”

Also, regarding the low number of PBMC samples stimulated with any given peptide, how did the authors define immunodominance for the purpose of this manuscript?

As mentioned above, we have increased the number of HLA-A:11:01-expressing participants to a total of n=24 in our study, and n=7 for peptide stimulations.

As mentioned in response to Reviewer 1, we hope the Reviewer appreciates that based on the global distribution of HLA-A*11:01 being at ~7.3%, we needed to HLA type ~328 participants at $AUD 220 (total of $AUD 72,160) for our study.

Part III – Minor Issues: Editorial and Data Presentation Modifications

Please use this section for editorial suggestions as well as relatively minor modifications of existing data that would enhance clarity.

Reviewer #1: Plots result often difficult to interpret:

1) Which donors have been used in each experiment in Fig. 2, 4, 5 and 7? In order to help readers interpret the plots, each donor should be shown with a different symbol and the donor code should be indicated in the legend. Did you use the same or different donors for the screenings showed in Fig. 2, 4, 5 and 7?

We thank the Reviewer for their comment. To more clearly show which donors were used in each experiment, we have indicated individual donors with different symbols and have included legends with each graph. Following the Reviewer’s comment, Figures 2, 4, 5 and 7 were modified.

2) In Fig 5 different symbols are used to distinguish between Indigenous and non-indigenous donors. This reviewer wonders whether this info is really relevant for the interpretation of the results.

While HLA-A*11:01 is frequently expressed in Indigenous Australians as highlighted in Figure 1, we agree that the main focus of this manuscript is epitope discovery rather than comparing immune responses in Indigenous and non-Indigenous Australians. Following the Reviewer’s comment, we have removed the different symbols representing these two population groups and have instead used different symbol for each individual donor.

3) How did the authors set the cut off of positive or negative response? The results are shown as % IFN-γ+ of CD8+ T cells, but what was the range of basal IFN-γ expression? Showing the “No Ag” condition as well as providing a description of the rationale for determining positivity would be helpful.

We thank the Reviewer for their comment. Indeed, immunogenicity is difficult to define as it can be confounded by an individuals’ HLA repertoire. To better define a positive immunogenicity readout, we have altered our analysis method. We previously subtracted the DMSO background IFN-γ response from each of the peptide conditions to determine immunogenicity, but without a clear cut-off. Following the Reviewer’s comment, we have modified our analyses to now compare the negative control condition (DMSO) and peptide stimulation conditions using a Friedman test with Dunn’s correction for multiple comparisons, or a Wilcoxon signed-rank test, where appropriate, to determine significant differences in the frequency of IFN-γ^+^CD8^+^ T cells. If stimulation with a certain peptide resulted in clear activation (above basal IFN-γ levels observed in the DMSO condition) in selected donors only, but was not determined to be significantly increased with statistics, we considered these donors as responsive, however in this instance we considered the peptide as subdominant rather than immunodominant.

Our analyses in Figure 2, 4, 5 and 7 now show statistical significance where appropriate.

4) Information about the antibody staining panels and gating strategy used to identify epitope-reacting CD8+ T cells is not provided in the text. This information should be added in the method section. Did you gate on CD8+ IFN-γ+ cells or only on IFN-γ+ total population? How did you define activated CD8+ T cells when showing the combination of cytokine expression (CD107a, IFN-γ, MIP1-b, TNF)?

Based on the Reviewer’s comment, we have added a gating strategy to Supplementary Figure 6 and added more details to the method for intracellular cytokine staining on page 19:

“***CD8^+^ T cell re-stimulation and intracellular cytokine staining****.* Intracellular cytokine staining (ICS) was performed on day 10-12 to identify epitope-specific CD8^+^ T cells after influenza virus or peptide stimulation. Virus-specific CD8^+^ T cells were re-stimulated with peptide-pulsed C1R-A*11:01 cells at a 1:2 ratio and antigen-specific CD8^+^ T cells were re-stimulated with 1 μM of individual or pooled influenza peptides. Re-stimulations were done for 5 h in the presence of brefeldin A (BD GolgiPlug), monensin (BD GolgiStop) and anti-CD107a-AF488 antibody. After stimulation cells were surface stained with LiveDead NIR fixable viability dye (Invitrogen), anti-CD3-PE-Cy7, anti-CD4-PE and anti-CD8-PerCP-Cy5.5 before permeabilizing for intracellular staining with anti-IFN-γ-V450, anti-TNF-AF700 and anti-MIP1-β-APC antibodies. The gating strategy for determining CD8^+^ T cell activation is shown in Supplementary Figure 6.”

5) When possible, statistical analysis should be applied and shown in the plots (e.g. Fig 5).

We have now included our negative control condition in order to perform paired t-tests in our analyses. This has been applied to Figures 2, 4 and 5, and updated results on pages 6, 7, 9 and 10. Additionally, paired t-tests have been performed between the HLA-A*02:01-restricted and HLA-A*11:01-restricted peptide stimulation conditions in Figure 7. We thank the Reviewer for this comment.

Reviewer #2: No comments

## References

1.	Koutsakos M, Illing PT, Nguyen THO, Mifsud NA, Crawford JC, Rizzetto S, et al. Human CD8+ T cell cross-reactivity across influenza A, B and C viruses. Nature Immunology. 2019;20(5):613-25.

2.	Nielsen M, Lundegaard C, Worning P, Lauemøller SL, Lamberth K, Buus S, et al. Reliable prediction of T-cell epitopes using neural networks with novel sequence representations. Protein Sci. 2003;12(5):1007-17.

3.	Andreatta M, Nielsen M. Gapped sequence alignment using artificial neural networks: application to the MHC class I system. Bioinformatics. 2016;32(4):511-7.

PLOS authors have the option to publish the peer review history of their article ([what does this mean?](https://journals.plos.org/plospathogens/s/editorial-and-peer-review-process#loc-peer-review-history)). If published, this will include your full peer review and any attached files.


If you choose “no”, your identity will remain anonymous but your review may still be made public.


Do you want your identity to be public for this peer review? For information about this choice, including consent withdrawal, please see our [Privacy Policy](https://www.plos.org/privacy-policy).

Reviewer #1: No

Reviewer #2: No

Figure Files:

While revising your submission, please upload your figure files to the Preflight Analysis and Conversion Engine (PACE) digital diagnostic tool, [https://pacev2.apexcovantage.com](https://pacev2.apexcovantage.com/). PACE helps ensure that figures meet PLOS requirements. To use PACE, you must first register as a user. Then, login and navigate to the UPLOAD tab, where you will find detailed instructions on how to use the tool. If you encounter any issues or have any questions when using PACE, please email us at [figures@plos.org](mailto:figures@plos.org).

Data Requirements:

Please note that, as a condition of publication, PLOS' data policy requires that you make available all data used to draw the conclusions outlined in your manuscript. Data must be deposited in an appropriate repository, included within the body of the manuscript, or uploaded as supporting information. This includes all numerical values that were used to generate graphs, histograms etc.. For an example see here on PLOS Biology: <http://www.plosbiology.org/article/info%3Adoi%2F10.1371%2Fjournal.pbio.1001908#s5>.

Reproducibility:

To enhance the reproducibility of your results, we recommend that you deposit your laboratory protocols in [protocols.io](http://protocols.io/), where a protocol can be assigned its own identifier (DOI) such that it can be cited independently in the future. Additionally, PLOS ONE offers an option to publish peer-reviewed clinical study protocols. Read more information on sharing protocols at <https://plos.org/protocols?utm_medium=editorial-email&utm_source=authorletters&utm_campaign=protocols>

We have ensured our figures meet PLoS Pathogens quality and have uploaded Source Data Excel file containing our raw data for all the figures. Mass spectrometry data will be made publicly available on the Proteomics Identifications Database (PRIDE) upon publication. Crystal structures have been deposited to the Protein Data Bank (PDB) and will be made publicly available upon publication.
